# Work-Related Upper Limb Disorders: A Case Report

**DOI:** 10.3889/oamjms.2015.033

**Published:** 2015-03-11

**Authors:** Zlatka Borisova Stoyneva, Svetlan Dermendjiev, Tihomir Dermendjiev, Hristo Dobrev

**Affiliations:** 1 *University Hospital St. Ivan Rilsky, Clinic of Occupational Diseases, Medical University - Sofia, Bulgaria*; 2 *University Hospital St George, Medical University - Plovdiv, Bulgaria*

**Keywords:** occupational disease, work-related disease, musculoskeletal upper limb disorders, occupational risk, connective tissue disease

## Abstract

In this study the complex interrelationship between physical factors, job stress, lifestyle and genetic factors on symptoms of work-related musculoskeletal disorders of the upper limbs is demonstrated by a case report and discussion of the literature. A 58 year old woman with long lasting complaints of the upper limbs with increasing intensity and duration, generalisation, combined with skin thickness, Raynaud’s phenomenon, joint disorders, arterial and pulmonary hypertension, metabolic lipid dysfunctions is presented. Occupational history proves continuous duration of service at a job with occupational physical static load with numerous repetitive monotonous systematic motions of fingers and hands as a weaver of Persian rugs followed by work at an automated loom and variable labour activities. Though the complaints dated since the time she was a manual weaver, the manifestations of generalized joint degenerative changes, system sclerosis with Raynaud’s phenomenon with similar upper extremities signs and symptoms discount upper limbs musculoskeletal disorder as caused only or mainly by occupational risk factors. The main principles and criteria for occupational diagnosis of musculoskeletal upper limb disorders and legislative requirements for their reglamentation are discussed.

## Introduction

Work-related upper limb disorders are the dominating musculoskeletal diseases in the general population [1-4], covering 24% of the working population within the European Union [5]. In Bulgaria they represent 69.5% of all registered cases with occupational diseases [6].

The apprehensions and attitudes about work-related and occupational diseases have been discussed for a long time and were separated since 1996. According to the definition of the World Health Organization an occupational disease is any disease contracted primarily as a result of an exposure to risk factors arising from work activity while work-related diseases have multiple causes, among which factors in the work environment may play a role, together with other risk factors [7]. In Bulgaria the legal definition of an occupational disease is similar, i.e. the disease caused only or mainly by occupational risk factors and included in the List of Occupational Diseases, while work-related disease is multifactorial in origin and occupational risk factors are not the only or main causes, but they provoke, aggravate, worsen or complicate the disease. The occupational disease is compensated for but the work-related one is not.

Physical work factors, psychosocial and environmental factors and individual characteristics affect work-related upper extremity disorders. Biomechanical risks as highly repetitive precision tasks, forceful manual exertions, sustained awkward postures of the upper limbs, and work organization and psychosocial factors as temporal pressure and stress, maximization of minor mistakes, job content and demands, perceived stress levels, decision latitude, low social support, low job control, satisfaction and monotonous work, shift work, insufficient recovery time, and fatigue are recognized as associated with these disorders [8-10]. The great social significance of work-related upper limb disorders results from their high incidence, from individual suffering and life quality decrease, heavy economic and security impacts when they affect work capacity and induce sickness absences, poorer work performance, reduced productivity, early retirement and permanent work disability [11, 12] leading to a significant direct cost burden on health care systems [13].

It is often difficult to prove and identify a disease as occupationally induced or work-related especially when it concerns musculoskeletal disorders associated with a variety of possible causal factors.

The objective of this study was to discuss the process of diagnosing a work-related musculoskeletal disease using the current criteria for accepting or rejecting the occupational origin presenting a case report.

## Case Report

A 58-year-old Caucasian female was hospitalized in the Department of Occupational Diseases in University Hospital St. George, Plovdiv for complaints in her hands and arms.

Her occupational history was based on given documents, containing descriptions of her jobs, their duration and exposure to occupational risk factors. The global length of patient’s service comprised 27 years, of which 20 years she had been a carpet-weaver in a factory for Persian rugs in a southern Bulgarian town. This occupation is characterized with systematic physical static load, numerous repetitive monotonous motions of fingers and hands at high rate, insufficient recovery time, forceful manual exertion, sustained awkward posture of the wrists, elbows or shoulders, temporal pressure and stress, maximization of minor mistakes, insufficient recovery time, and fatigue. The main part of this occupation with duration of 16 years she had been weaving by hand, and the rest 4 years afterwards she had weaved at an automated loom, both of them being with overstrain hazards that could cause occupational or work-related upper limb disorders. Her next job was as a common worker for the last 7 years with variable movements. At the period of her hospitalization she had retired since 14 months.

### Present medical history

Since 15 years she had gradually increasing in duration and level of acuteness complaints of pains in the neck and shoulder girdle muscles propagating distally along her forearms, hands, interphalangeal joints, accompanied by tingling and numbness of the hands and fingers, stiffness of the fingers and rigidity of her neck. At the beginning they were rare, usually during nights and early in the mornings but lately they became almost permanent. Her manual dexterity and strength had gradually decreased losing hold of objects. Since several years the skin color of some fingers and/or phalanges rapidly changed turning blanched or blue provoked by cold. Three years ago she was diagnosed with progressive systemic sclerosis on the basis of joint pains of her hands and also of her legs and Raynaud’s phenomenon manifestations with paroxysmal cold-induced clearly demarcated skin color changes of the fingers, thickness of the finger and forehead skin. Systematic medication with *D*-penicillamine and vasodilators were prescribed. Nevertheless periods of acute pains of the small joints of the hands and back, especially low back, cyanosis and paresthesia of the fingers and toes reoccurred and manifested.

### Past medical history

Arterial hypertension since 18 years, nephrolithiasis, post menopausal osteoporosis, no fractures and special treatment applied.

**Figure 1:**
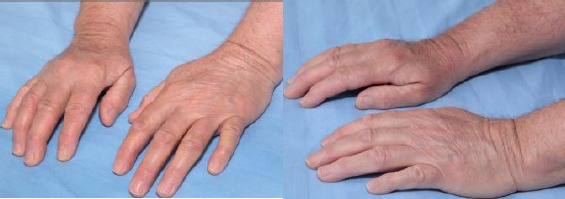
*Patient’s hands*.

By physical examination there were white skin thickened on the forehead, cheeks, forearms and fingers, pink mucous membranes, well-developed, well nourished in no apparent distress. Thyroid gland was diffusely enlarged. Extremities: cold and cyanotic skin of the fingers, sclerodactylia; tender to palpation wrists, 2-nd and 3-rd metacarpophalangeal joints; joint stiffness, reduced range of motion with embarrassed flexion of metacarpophalangeal, proximal and distal interphalangeal joints, decreased grip strength, impossible full extension of metacarpophalangeal and proximal interphalangeal joints; Heberden’s nodes; interphalangeal deformities (Fig. 1).

Neurological examination revealed vertebral cervical syndrome with rigid muscular tone and decreased range of motions, tender points of Erb and Vallaix bilaterally, hypesthesia along C6 dermatomes, erythrocyanotic fingers, positive Laignel-Lavastine test.

### Laboratory results

Immunological investigations are presented at Table 1.

**Table 1:** Immunological investigations.

Type	Method	Results	Reference ranges	
Immunoglobulines			
IgE	ELISA	<25 IU/ml	<25 IU/ml
IgG	ELISA	15.250 g/l	6.58-18.37 g/l
IgM	ELISA	0.393 g/l	0.40-2.63 g/l
IgA	ELISA	1.611 g/l	0.71-3.60 g/l
Autoantibodies			
Antinuclear antibodies (ANA)	Fluorescence technique	negative	<1:40
Anti-topoisomerase I (Anti Scl 70)	ELISA	0.12	< 0.15 IU/ml
Anti-nDNK	ELISA	negative	<25 IU/ml
Antineutrophil cytoplasmic antibodies (ANCA)	Myeloper-oxidase-MPO, ELISA		<5 U/ml
Antiphospholipid antibodies (APLAs), i.e. subgroup Anticardiolipin antibodies (ACAs)	ELISA	3.3 IU/ml	<10GP U/ml
Rheumathoid factor (RF)	IgM – ELISA	3.3 IU/ml	< 20 IU/ml/
Cryoglobulins	Qualitative analysis	Negative	Negative

Electroneurography revealed decreased anterior spinal root conduction C6, C7 bilaterally, more expressed for C6 and on the left.

**Figure 2:**
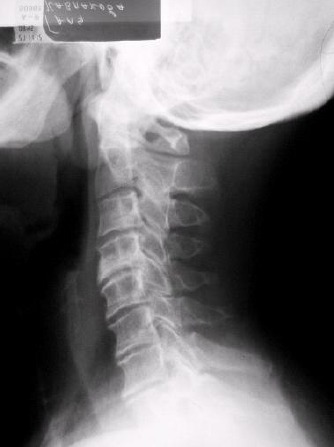
*X-ray of the cervical vertebrae in a profile projection*.

Radiological examinations are presented at Table 2 and Fig. 2.

**Table 2:** Radiological examinations.

Radiological examination of	Findings
Cervical spine	Osteochondrosis C4-5, C5-6, C6-7 and C7-Th1, spondylosis, straightening of cervical lordosis

Lungs and heart	Pulmofibrosis, congestion of the hiluses

Wrists and fingers	arthroso-arthritis with narrowed joint clefts of the interphalangeal and metacarpophalangeal joints

Hip joints	Bilateral coxarthrosis

Knee joints	Bilateral gonarthrosis more expressed on the right

**Figure 3:**
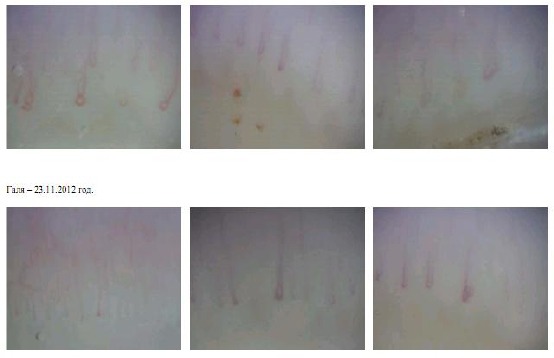
*Nailfold capillarographies before (the first three pictures) and after a three and a half months treatment (the next three pictures)*.

Nailfold capillaroscopy: In the first three capillarography immages irregular nailfold capillaries with avascular zones, many spastic and corkscrew-like, folded, dilated capillaries in both arterial, venular parts and a-v transition with a tendency to aneurysms, extravascular hemorrhages, pericapillary oedema and light halo on a pale yellowish phone were seen. The next capillarographies with fewer changes made three and a half months later after active treatment were similar but with prevailing spastic capillaries and single dilated, aneurysmatic capillaries without hemorrhages (see Fig. 3).

Handgrip dynamometry: significantly decreased muscular strength of both hands.

ECG – left type left ventricular hypertrophy, ST-segment depression in V5-6.

Doppler echocardiography - left ventricular hypertrophy, pump dysfunction - ejection fraction 53%, fractional shortening 27% and stroke volume 4-1 ml. Pulmonary arterial hypertension – augmentation pressure 36 mm. Right ventricular dysfunction.

### Consultations

**Neurologist:** Cervical disc disease with secondary radiculopathy C6, C7 with sensory and autonomic vascular dysfunction in the upper limbs.

**Rheumatologist:** Progressive systemic sclerosis – limited form, Raynaud’s phenomenon without immunological and clinical activity. Arterial hypertension II stage, moderate degree without heart deficiency. Hypertensive heart disease. Pulmonary arterial hypertension. Right ventricular and pump dysfunction. Hypercholesterolemia. Post menopausal osteoporosis without pathological fractures. Nephrolithiasis.

**Specialist in occupational diseases:** Cervical radiculopathy C6, C7 with predominantly sensory and autonomic dysfunctions with ischemic Raynaud’s type attacks with complex etiology – related to degenerative cervical vertebral changes, autoimmune connective tissue disease, neurocirculatory dysfunction accompanying arterial hypertension and metabolic disorders and provoked and aggravated by work-induced repetitive overstrain.

### Diagnostic data

She was diagnosed with progressive systemic sclerosis (limited form), Raynaud’s phenomenon without immunological and clinical activity. Arterial hypertension II stage, moderate degree without heart deficiency. Cor hypertonicum. Hypertensive heart disease. Pulmonary arterial hypertension. Right ventricular and pump dysfunction. Hypercholesterolemia. Post menopausal osteoporosis without pathological fractures. Nephrolithiasis. Recurrent depressive disorder. Cervical spondylosis and osteochondrosis C4-5, C5-6, C6-7 et C7-Th1 and secondary cervical radiculopathy C6 and C7 with sensory and autonomic dysfunctions.

## Discussion

Work-related upper limb disorders include a wide range of diseases with manifestations of pain, functional and sensory impairments of the neck, shoulders, elbows, forearms, wrists and hands, and are a significant problem within the European Union with respect to ill health, productivity and associated costs.

The study presents a case report of a female patient with upper limb disorders. She has worked as a carpet-weaver for a long period of time. This is a typical job with proved static and dynamic overstrain of the upper limbs and forced monotonous repetitive movements of the fingers and hands [14], causing occupational upper limb disorders [15, 16]. The presented case has experienced complaints typical for occupationally induced upper limb disorders. Nevertheless she was diagnosed not with occupational but with work-related secondary to osteochondrosis and spondylosis cervical radiculopathy with sensory and autonomic dysfunctions. The degenerative cervical vertebral disorders were a part of proved polyarthrosis in a patient above 40 years of age but not isolated to the mostly occupationally overstrained joints. Besides that she has progressive systemic sclerosis with secondary Raynaud’s phenomenon. Though the occupational history revealed continuos work with biomechanical risks for the upper limbs as a carpet weaver (the minimal exposure period according to our law being six years), the subsequent retirement for more than a year (only one year free of occupational risk factors period allowed for registration of such occupational disease in Bulgaria) did not improve her complaints, i.e. negative elimination test. Her upper limb disorders are not predominantly occupationally induced, but are multifactorial due to age, polyarthrosis, connective tissue autoimmune disease, and also to the provoking and aggravating occupational risk factors, i.e. work-related.

The important information and detailed findings of a patient can often be lost in research studies where single results are aggregated [17]. In the era of evidence-based practice, we need practice- and legal-based evidence for our decisions and conclusions on the ground of: a detailed history, including occupational and comorbidity one; thorough examinations and investigations; world’s medical knowledge and contemporary clinical views and concepts.

The limitations of our study to present a single clinical case with definite co-morbidities and an exact job connected with primarily biomechanical occupational risk factors for upper limb disorders diagnosed by the criteria of a distinct European country influence the interpretation of the findings, limit the scope of analysis and discussion, constraint on generalizability.

Further high methodological quality studies are needed to better understand and prove the causal relationship between risk factors and work-related musculoskeletal disorders, define and harmonize the criteria for their diagnosis.

In conclusion, work-related upper limb disorders cause substantial discomfort, movement dysfunction, disability and loss of productivity of the worker. Many of these diseases with a multifactor aetiology may be occupationally induced only under certain conditions. Therefore the final assessment of the disease origin requires the common efforts of different specialists, including such in occupational medicine with competences about occupational risk factors, valid laws, principles of diagnostics and criteria for an appraisal of the occupational character of a disease.
